# Severity of Coronary Artery Diseases Among Pre- and Postmenopausal Women With Acute Coronary Syndrome: A Hospital-Based Study in Bangladesh

**DOI:** 10.7759/cureus.50514

**Published:** 2023-12-14

**Authors:** Ayesha Siddika, Fazila-Tun-Nesa Malik, Md Kalimuddin, Nahidul Hasan, Nazir Ahmed, Mohammad Badiuzzaman, Mir Nesaruddin Ahmed, Ashok Dutta, Mir Ishraquzzaman, Md. Shamim Chowdhury

**Affiliations:** 1 Internal Medicine, Bangladesh College of Physician and Surgeon, Dhaka, BGD; 2 Cardiology, National Heart Foundation Hospital & Research Institute, Dhaka, BGD

**Keywords:** acute coronary syndrome, clinical features, coronary angiographic severity, gensini score, premenopausal and postmenopausal women

## Abstract

Background: Postmenopausal women present with more severe coronary artery disease (CAD) in addition to multiple comorbidities. However, there are limited data available to compare the risk factors, clinical characteristics, and angiographic severity of CAD between pre- and postmenopausal women with the acute coronary syndrome (ACS).

Aim: This study aimed to assess and compare the severity of CAD in pre- and postmenopausal women with ACS.

Methods: This cross-sectional observational study was conducted at the Department of Cardiology of NHFH RI. A total of 140 female patients with ACS were enrolled and then divided into Group I (premenopausal) and Group II (postmenopausal) on the basis of menopause history. Clinical data and coronary angiographic severity were compared between both groups.

Results: The mean age of the premenopausal group was 41.53 ± 5.45 years, and that of the postmenopausal group was 57.23 ± 7.45 years. Family history of premature CAD was significantly more common in the premenopausal group than in the postmenopausal group (35(50%) vs. 23(32.9%); p=0.017)). DM and smokeless tobacco were more prevalent in the postmenopausal group (48(68.6%) vs. 28(40%); p=0.001 and 14(20%) vs. 2(2.9%); p=0.002). Atypical presentation was more common in the premenopausal group (21(30%) vs. 9(12.9%); p=0.013). Most of the patients in both groups presented with unstable angina followed by NSTEMI and STEMI. Mean left ventricular ejection fraction was lower in the postmenopausal group than in the premenopausal group (50.71 ± 8.38% vs. 53.74 ± 7.46%; p=0.026). Normal coronary angiogram and single-vessel disease were more prevalent in the premenopausal group (22(31.4%) vs. 12(17.1%); p=0.04) and (22(31.4%) vs. 11(15.7%); p=0.002), whereas triple-vessel disease was more prevalent in the postmenopausal group (34(48.6% vs. 14(20%); p=0.001). The left anterior descending artery was the most commonly involved vessel in the postmenopausal group (67(95.7%) vs. 60(85.7%); p=0.04). Finally, the mean Gensini score was higher in the postmenopausal group than in the premenopausal group (56.1 ± 43.4 vs. 33.5 ± 36.9; p=0.001).

Conclusion: Family history of premature CAD and atypical presentation were common in premenopausal ACS patients. DM and smokeless tobacco use were more prevalent in the postmenopausal group than in the premenopausal group. Normal coronary angiogram and single-vessel disease were more prevalent in the premenopausal group, and triple-vessel disease was more common in the postmenopausal group. CAD was more severe in the postmenopausal group.

## Introduction

The incidence of coronary artery disease (CAD) in women older than 65 years is similar to that in men and even surpasses that in men after 75 years [1]. Young women suffer less from heart diseases due to the vascular protective action of estrogen, which helps in preventing atherosclerosis. During menopause, a woman’s estrogen levels drop to approximately one-third of those during pre-menopausal years. The primary causes of morbidity and death for women in both developed and developing nations are ischemic heart disease and cerebrovascular events (i.e., strokes), which are exacerbated by changes in the synthesis of female hormones during menopause.

Recognition of prodromal symptoms was reported to be critical for preemptive coronary heart disease screening, and effective diagnosis and treatment. At the time of presentation of acute coronary syndrome (ACS), 96% of women experience prodromal symptoms, with unusual fatigue (73%) and sleep disturbance (50%) being the most common symptoms [2]. It is important to recognize the symptoms that are associated with acute subsequent cardiac events. However, symptoms among younger women are atypical, and patients with silent myocardial ischemia usually have more extensive and severe diseases.

Coronary angiographic profiles reveal a distinct difference in the pattern of CAD between premenopausal and postmenopausal women, with a greater incidence of angiographically normal epicardial coronaries in the former, suggesting a non-atherosclerotic pathology. Even in those with significant coronary lesions, single-vessel disease is more common among premenopausal women, whereas among postmenopausal women, multi-vessel disease is the norm, with the majority having three-vessel disease [3]. Gensini and Friesinger scores, which are used to assess the severity of CAD, are also significantly higher in postmenopausal women [4].

Compared to premenopausal women with ACS, postmenopausal women with ACS tend to have more vulnerable culprit lesions. Moreover, characteristics of vulnerable plaques, such as thin-cap fibroatheroma, lipid-rich plaque, large lipid arc, longer lipid length, macrophage accumulation, microchannel, and cholesterol crystals, are more common in postmenopausal women [5]. Postmenopausal and premenopausal women also differ in the symptoms, risk factors, disease characteristics, prognosis, and recurrence of CAD. However, due to the low incidence of CAD in premenopausal women, research on this particular population remains insufficient [6]. Therefore, this study aimed to investigate the complete sociodemographic characteristics, risk factors, clinical presentation, coronary angiographic profile, vessel score, and Gensini score of pre- and postmenopausal women with ACS.

## Materials and methods

This observational, cross-sectional study was performed at a medical teaching hospital in the central region of Bangladesh. Institutional ethical approval was obtained from the Ethics Review Committee of National Heart Foundation Hospital and Research Institute (Post Graduate Research Ref No. N.H.F.H.& R.I 4-14/7/Ad/04, dated December 28, 2021) before enrolling subjects in the study. Written informed consent was obtained from all study participants. The inclusion criteria were pre- and postmenopausal women with ACS who underwent coronary angiogram while admitted to the Department of Cardiology of National Heart Foundation Hospital and Research Institute, Dhaka, Bangladesh, between December 2021 and November 2022. The exclusion criteria were those with associated valvular heart diseases, congenital heart diseases, cardiomyopathy, and extremely severe concomitant diseases (e.g., severe dementia and advanced malignancy), as well as those who were unwilling to participate in the study.

Postmenopause was defined as a lack of menstrual bleeding for 12 months or a history of hysterectomy [7]. Premenopause was defined as not having experienced menopause or oophorectomy [8].

Patients were categorized into two groups according to menopausal status. Group I comprised premenopausal women with ACS, and Group II compromised postmenopausal women with ACS. ACS was diagnosed in patients consistent with a compatible clinical presentation and was further confirmed by electrocardiogram. Cardiac biomarker testing was done to diagnose the subtype of ACS. Complete sociodemographic characteristics, risk factors, clinical presentation, coronary angiographic profile, vessel score, and Gensini score were recorded in this study. Gensini score, which was first described in 1975 by Goffredo G. Gensini, is a widely used angiographic scoring system for quantifying the severity of CAD; it considers the geometrical severity of the lesion, cumulative effects of multiple obstructions, and the significance of the affected myocardium. Gensini score considers three main parameters for each coronary lesion: severity score, region multiplying factor, and collateral adjustment factor. First, a non-linear score is assigned to each lesion according to the reduction of the lumen diameter. A multiplier is then applied to the lesions depending on the functional significance of the area supplied by that segment. The final score is the sum of the lesion scores. Thus, Gensini Score = severity score × segment location multiplying factor × collateral adjustment factor. In particular, a lesion is defined as significant when it causes a ≥1% reduction in luminal diameter by visual assessment. The relative severity of the lesion is indicated using a score of 1 for 1%-25% obstruction and doubling that number as the severity of the obstruction progresses. For example, reductions of 25%, 50%, 75%, 90%, 99%, and complete occlusion are given Gensini scores of 1, 2, 4, 8, 16, and 32, respectively. In addition, the Gensini score considers the typical blood flow to the left ventricle in each conduit or section of a vessel, taking into account the differences between the left and right dominant coronary systems. Depending on where a lesion is located in the coronary tree and the functional significance of the area it supplies, a multiplier is given to each lesion score. A collateral adjustment factor is utilized-and the adjustment is decreased by the degree of illness in the vessel that is the source of collaterals if a segment is fully occluded or 99% stenosed and receiving collaterals. The total of all the lesion scores determines the final Gensini score [8].

Statistical analysis

The sample size was calculated using Gpower version 3.1.9.4, where the mean difference between two independent means (two groups) was applied, taking effect size as 0.50, alpha error as 0.05, power as 80%, and location ratio as 1. The total sample size was 140. Data entry and all statistical analyses were performed using SPSS version 22 (IBM Corp., Armonk, NY). Categorical data were reported in terms of percentage and frequency, and continuous variables were reported in terms of mean±standard deviation (SD).

Means of continuous variables were compared using Student’s t-test. Associations between categorical variables were evaluated using the chi-squared test. Correlation studies of continuous variables were conducted using Pearson’s correlation coefficient. A p-value of <0.05 (with 95% confidence interval) indicated statistical significance.

## Results

A total of 140 cases of ACS diagnosed in the Department of Cardiology from December 2021 to November 2022 were included in this study. The mean ages of Group I and Group II were 41.53 ± 5.45 years and 57.23 ± 7.45 years, respectively. The largest age group in Group I was 41-45 years (45.7%, n=32), whereas that in Group II was 56-60 years. Among the respondents, hypertension, dyslipidemia, family H/O premature CAD, and diabetes mellitus (DM) were the most common risk factors in both groups. No statistically significant differences in hypertension and dyslipidemia incidence were observed between the two groups (p>0.05). However, DM and smokeless tobacco were more prevalent in Group II (p<0.05). Family H/O premature CAD was significantly more common in Group I, as was oral contraceptive pill (OCP) intake. Most of the patients in Group I were obese (58.6%, n=41), followed by normal (25.7%, n=18), and overweight (15.7%, n=11). Similarly, in Group II, most patients were obese (50.0%, n=35), followed by overweight (28.6%, n=20), normal (20%, n=14), and underweight (1.4%, n=1). Analysis revealed that there were no statistically significant differences between the two groups (p>0.05) (Table 1).

**Table 1:** Baseline demographic characteristics of the study population (n=140). HTN=hypertension, DM=diabetes mellitus, SD=standard deviation, OCP=oral contraceptive pill, BMI=body mass index, CAD=coronary artery disease

Demographic characteristics		Group I n=70 n(%)	Group II n=70 n(%)	p-value
Age (year)	≤35	12 (17.1)	0 (0.0)	0.001
	36–40	16 (22.9)	0 (0.0)	0.001
	41–45	32 (45.7)	3 (4.3)	0.001
	46–50	10 (14.3)	10 (14.3)	0.805
	51–55	0 (0.0)	20 (28.6)	0.001
	56–60	0 (0.0)	21 (30.0)	0.001
	>60	0 (0.0)	16 (22.9)	0.001
	Mean ± SD	41.53 ± 5.45	57.23 ± 7.45	0.001
Risk factors	HTN	52 (74.3)	58 (82.9)	0.217
	DM	28 (40.0)	48 (68.6)	0.001
	Dyslipidemia	30 (42.9)	36 (51.4)	0.310
	Family H/O premature CAD	35 (50.0)	23 (32.9)	0.017
	OCP	38 (54.3)	0(0.0)	0.001
	Smokeless tobacco	2 (2.9)	14 (20.0)	0.002
	Smoking	0 (0.0)	0 (0.0)	
	Underweight	0 (0.0)	1 (1.4)	0.316
	Normal	18 (25.7)	14 (20)	0.421
BMI (kg/m^2^)				
	Overweight	11 (15.7)	20 (28.6)	0.067
	Obese	41 (58.6)	35 (50.0)	0.309
	Total	70 (100.0)	70 (100.0)	
	Mean ± SD	26.43 ± 4.28	25.70 ± 3.78	0.282

Among the study population, clinical presentation other than angina pain revealed that atypical presentation (e.g., palpitation, epigastric pain, tingling and numbness sensation in the limbs, and feelings of uneasiness) was common in Group I, whereas shortness of breath was common in Group II. There was a statistically significant difference in atypical presentation between the two groups (p<0.05). Clinically, it was evident that in Group I the majority of patients presented with unstable angina (58.6%), followed by non-ST-elevation myocardial infarction (NSTEMI) (30%) and ST-elevation myocardial infarction (STEMI) (11.4%), whereas in Group II, these rates were 42.9%, 37.1%, and 20.0%, respectively. Analysis revealed that there was no statistically significant difference in subsets of ACS between the two groups (p>0.05) (Table 2).

**Table 2:** Clinical characteristics of the study patients (n=140). STEMI=ST-segment elevation myocardial infarction, NSTEMI=non-ST-segment elevation myocardial infarction, UA=unstable angina

Clinical characteristics		Group I (n=70) n (%)	Group II (n=70) n (%)	p value
Clinical presentation	Shortness of breath	12 (17.1)	21 (30.0)	0.073
	Atypical presentation	21 (30.0)	9 (12.9)	0.013
Clinical diagnosis	STEMI	8 (11.4)	14 (20.0)	0.164
	NSTEMI	21 (30.0)	26 (37.1)	0.371
	UA	41 (58.6)	30 (42.9)	0.063
	Total	70 (100.0)	70 (100.0)	

The mean values of fasting blood sugar (FBS), HbA1C, and serum creatinine were higher in Group II than in Group I. There were statistically significant differences in the mean values of HbA1c and serum creatinine between the two groups (p<0.05), whereas there was no statistically significant difference in that of FBS (p>0.05). The mean values of troponin-I and creatine kinase myocardial band (CKMB) were also higher in Group II than in Group I, but these differences were not statistically significant (p>0.05). The mean value of HDL-C was lower in Group II than in Group I, and this difference was significant (p<0.05) (Table 3).

**Table 3:** Comparison of biochemical and echocardiographic variables between the groups (n=140). FBS=fasting blood sugar, HbA1C=Glycated hemoglobin, CKMB=creatine kinase myocardial band, TG=triglycerides, HDL=high-density lipoprotein, LDL=low-density lipoprotein, TC=total cholesterol, LVEF=left ventricular ejection fraction.

Variables		Group I (n=70)	Group II (n=70)	p-value*
Lab investigation	FBS (mmol/L)	6.8 ± 2.4	7.3 ± 2.2	0.232
	HbA1C (%)	6.8 ± 1.7	7.7 ± 2.0	0.008
	Serum creatinine (mg/dL)	0.9 ± 0.1	1.1 ± 0.4	0.001
	CKMB (U/L)	38.0 ± 39.6	42.3 ± 23.8	0.470
	Troponin-I (ng/mL)	7.7 ± 22.6	17.2 ± 59.7	0.225
	TG (mg/dL)	127 ± 57.2	144.7 ± 68.9	0.118
	HDL(mg/dL)	40.5 ± 10.2	35.3 ± 6.0	<0.001
	LDL (mg/dL)	105.1 ± 26.8	115 ± 38.4	0.066
	TC (mg/dL)	156.4 ± 44.1	166.2 ± 57.3	0.258
	≤30	1 (1.4)	0 (0.0)	
	31–44	7 (10.0)	16 (22.9)	
LVEF (%)				
	45–54	27 (38.6)	27 (38.6)	
	>55	35 (50.0)	27 (38.6)	
	Total	70 (100.0)	70 (100.0)	
	Mean ± SD	53.74 ± 7.46%	50.71 ± 8.38%	0.026

The severity of CAD in the study patients was assessed in terms of the number of coronary artery involvement. In Group I, 31.4% (n=22) of patients had single-vessel disease, 17.1% (n=12) had double-vessel disease, and 20% (n=14) had triple vessel disease, whereas in Group II, these rates were 15.7% (n=11), 18.6% (n=13), and 48.6% (n=34), respectively. Single-vessel disease was more prevalent in premenopausal women (p<0.002), and triple-vessel disease was more prevalent in postmenopausal women (p<0.001). The normal vessel was also significant regarding the site of coronary artery lesions of RCA, LCX, and LAD vessels were normal more in Group I, and proximal involvement was more common in Group II, with these findings being statistically significant (p<0.05). LMCA was involved in 18.6% (13) of patients in Group I and 15.7% (11) of patients in Group II, but this finding was not statistically significant and more prevalent in premenopausal women (Table 4).

**Table 4:** Severity of coronary artery disease in the study population by number of coronary artery involvement (n=140). Single vessel disease, double vessel disease, triple vessel disease, right coronary artery, left circumflex artery, left anterior descending artery, left main coronary artery.

Characteristics		Group I (n=70) n (%)	Group II (n=70) n (%)	p-value
Number of involved vessels	None	22 (31.4)	12 (17.1)	0.049
	SVD	22 (31.4)	11 (15.7)	0.002
	DVD	12 (17.1)	13 (18.6)	0.825
	TVD	14 (20.0)	34 (48.6)	0.001
Number of coronary artery involvement	RCA	55 (78.6)	63 (90.0)	0.063
	LCX	55 (78.6)	55 (78.6)	1.00
	LAD	60 (85.7)	67 (95.7)	0.042
	LMCA	13 (18.6)	11 (15.7)	0.564
Site of coronary artery lesion	RCA[A1]			
	Normal	24 (43.7)	7 (11.0)	0.001
	Proximal	19 (34.5)	43(68.3)	0.001
	Mid	8 (14.5)	10 (15.9)	0.841
	Distal	4 (7.3)	3 (4.8)	0.565
LCX				
	Normal	25 (47.2)	11 (21.0)	0.006
	Proximal	22 (41.5)	33 (64.7)	0.018
	Mid	1 (1.9)	2 (3.9)	0.535
	Distal	5 (9.4)	5 (9.8)	0.949
	OM	2 (2.9)	4 (5.7)	0.404
LAD				
	Normal	23 (39.7)	9 (15.5)	0.004
	Proximal	35 (58.3)	49 (76.6)	0.003
	Mid	1 (1.7)	6 (9.4)	0.063
	Distal	1 (1.7)	0 (0.0)	0.300
	Diagonal	0 (0.0)	3 (4.3)	0.800
LMCA				
	Normal	57 (81.4)	59 (84.3)	0.654
	Disease	13 (18.6)	11 (15.7)	0.654

**Figure 1:**
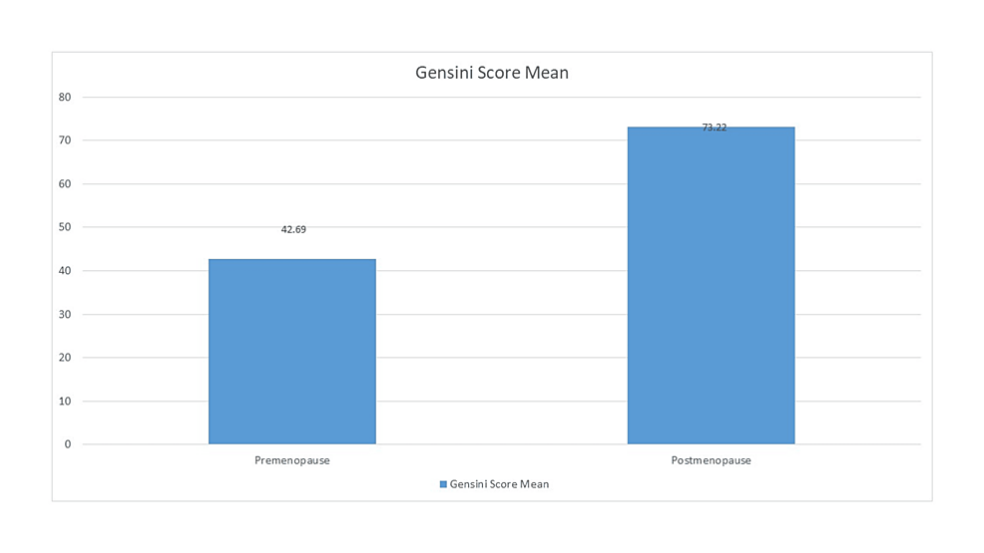
Comparison of Gensini score between the two groups with atypical presentation.

Among the respondents, mean Gensini score was 33.5±36.9 in Group I and 56.1±43.4 in Group II, which was a statistically significant difference (p<0.05) (Table 5, Figure 1).

**Table 5:** Severity of coronary artery disease in the study patients in terms of Gensini score (n=140). SD=standard deviation.

Variable	Group I (n=70)	Group II (n=70)	p-value
Gensini score			
Mean ± SD	33.5 ± 36.9	56.1 ± 43.4	
Median	21	56	
Mean Rank 58.6 82.4 0.001			

## Discussion

In this study, the mean ages of patients in Group I and Group II were 41.53 ± 5.45 years and 57.23 ± 7.45 years, respectively. Most of the patients in Group I belonged to the 41-45 years age group, whereas most in Group II belonged to the 56-60 years age group; these results agreed with those of a study done by Ahmed et al., who found mean ages of 41.6 ± 3.8 years and 56.0 ± 7.2 years in their study, respectively [4].

Regarding risk factors, hypertension, DM, dyslipidemia, family H/O premature CAD, and obesity were the most common in both groups. Smoking was not identified in either group, as smoking is a rare occurrence in female patients in our country. However, DM and smokeless tobacco were significantly more common in postmenopausal women. Prevalence of DM also increased with age and also long-standing uncontrolled DM was an important risk factor for multi-vessel coronary artery involvement and diffuse disease. Smokeless tobacco intake is more common in our country, especially in rural areas. Family history of premature CAD and OCP intake were significantly more common in premenopausal women. Ahmed et al., who performed a study on pre- and postmenopausal women with ACS in Bangladesh [4], found similar risk factor types in their work, namely dyslipidemia, obesity, smoking, DM, hypertension, and a family history of premature CAD. Hypertension was also a common risk factor in 76% of patients. A different study done by Islam et al. [9] showed an association between smokeless tobacco intake and CAD, which agreed with our results.

Diabetes carries a greater risk in female patients, as it is associated with platelet abnormalities, endothelial dysfunction, and negating the protective effects of estrogen [3]. Many recent studies have also shown that hypertension and DM are the two major risk factors for CAD in women [3,10-12]. The proportion of patients with none of the above conventional risk factors and still having CAD was significantly higher in the premenopausal group, signifying that there are factors other than conventional ones contributing to CAD in young women [3].

Our results showed that shortness of breath and atypical presentation (e.g., palpitation, epigastric pain, tingling and numbness sensation in the limbs, and feelings of uneasiness) were significantly more common in premenopausal patients, which matched the results of a previous study by Yihu et al. [11]. Unstable angina was the most common diagnosis in both groups, followed by NSTEMI and STEMI. This finding may be the result of coronary vasospasm being common in premenopausal women due to hormonal protection at reproductive age, as seen in previous studies [3]. Mean values of HbA1c and serum creatinine were significantly higher in the postmenopausal group, and HDL-C was lower, which may be due to the multiple comorbidities and lack of hormonal protection with progressively increasing age. In menopause, lipid metabolism is significantly affected, which is mainly manifested by an increase in LDL-C and a decrease in HDL-C [11].

The mean left ventricular ejection fraction (LVEF) of Group II patients was lower than that of Group I patients. LVEF decreased with advancing age in those with ACS. Postmenopausal patients suffer more from STEMI and NSTEMI than premenopausal patients, which may affect the reduction of LVEF in postmenopausal patients. Premenopausal women have significantly more normal coronary angiogram and SVD results, which may be due to the higher incidence of microvascular dysfunction in premenopausal women. TVD was significantly more common in postmenopausal women due to DM, increased age, and more AMI, as reported in other studies [4].

The number of coronary artery involvement in premenopausal women demonstrated more frequent single-vessel involvement (24% vs. 52%), whereas triple-vessel involvement was more common in postmenopausal women (12% vs. 40%, p<0.05). The coronary artery lesion site distribution in Group I and Group II revealed that the left anterior descending artery was most commonly involved, followed by the right coronary artery and the left circumflex artery. Left main coronary artery involvement was more common in Group I than in Group II. Differences in the involvement of the right coronary artery and the left anterior descending artery were highly statistically significant between the two groups (p<0.001).

In Group I, proximal LAD involvement was more common. In Group II, in addition to proximal LAD involvement, proximal LCX, and proximal and mid-RCA involvement were also common. At present, there is no authoritative statement about why LAD disease occurs more often in premenopausal women. Taking the anatomical structure of the left anterior descending artery into account, it is more active, and the left ventricle must consume more oxygen and nutrients. The anterior descending artery is more easily involved, as it is the main blood supply of the left ventricle, and it supports large areas of the ventricle. In their study, Ahmed et al. showed that proximal left anterior descending artery (LAD) lesions were the most common in the premenopausal group (56%), followed by RCA (36%) and LCX (30%). Our study found similar results, as the most common lesion in premenopausal women was LAD, followed by LCX and RCA. Similar findings were found by Ke-fei et al. [13], with the involvement of LAD, LCX, and RCA at 50.4%, 39.1%, and 40.7%, respectively. Vessel score indicated that single-vessel involvement was most common in Group I, whereas triple-vessel involvement was most common in Group II. Statistically significant differences in SVD and TVD were found between the two groups (p<0.05). In terms of severity assessment, the average Gensini score was 33.5± 36.9 in Group I and 56.1±43.4 in Group II, and this difference was highly statistically significant (p<0.001).

The Gensini and vessel scores of patients were much lower, which meant the lesions were confined mostly to one or two blood vessels. This result was most likely because young women are more likely to have inflammation, coronary spasm, plaque erosion, or rupture, and spontaneous coronary artery dissection. On the other hand, the clinical manifestations of postmenopausal women were more complex, in which collateral circulation was more easily formed, as they had a longer disease duration and long-term progression of the disease course. Many studies have also shown that postmenopausal women have higher CAD severity compared to premenopausal women, as evidenced by vessel score and Gensini score [4,13].

There are several limitations of our study. The study was conducted at a public hospital in Dhaka city, in which the respondents were self-selected purposively; therefore, it cannot be assumed that this sample is representative of the entire population of Bangladesh, as results may differ in different socio-demographic or cultural situations. Furthermore, in this study, the in-hospital outcome was not considered. Women with ACS who underwent CAG were included in the study, which may inadequately reflect the angiographic profile of women in the region. Finally, patient grouping was done on the basis of menstrual history, which may have been inadequate.

## Conclusions

Our results showed that the risk of ACS in women is low but not uncommon during the premenopausal period. Hypertension, DM, family history of premature CAD, and dyslipidemia were the most common risk factors for ACS in both groups, and OCP intake was also common in the premenopausal group. Smokeless tobacco intake was common in the postmenopausal group.

Coronary angiographic findings revealed less severe lesions and higher single-vessel involvement (most commonly LAD involvement) in premenopausal women compared to postmenopausal women. However, LM involvement and multiple-vessel involvement were found in the case of a high-risk group. A large-scale community-based study should be carried out to obtain more information. Social awareness regarding primary and secondary prevention, early diagnosis, and treatment are the keys to reducing the morbidity, mortality, and burden of CAD.
